# Modified Nutrition Risk in Critically Ill (mNUTRIC) Score to Assess Nutritional Risk in Mechanically Ventilated Patients: A Prospective Observational Study from the Pakistani Population

**DOI:** 10.7759/cureus.3786

**Published:** 2018-12-27

**Authors:** Hafiz Muhammad Ata ur-Rehman, Wasib Ishtiaq, Muhammad Yousaf, Sheher Bano, Abdul Malik Mujahid, Aftab Akhtar

**Affiliations:** 1 Internal Medicine, Shifa International Hospital, Islamabad, PAK; 2 Internal Medicine, Bolan University of Health and Medical Sciences, Quetta, PAK; 3 Dermatology, Jinnah Hospital - Allama Iqbal Medical College, Lahore, PAK

**Keywords:** mechanically ventilated patients, nutrition risk in critically ill score, nutritional assessment

## Abstract

Purpose

Typical nutritional assessment criteria and screening tools are ineffective in mechanically ventilated patients who are often unable to report their food intake history. The Nutrition Risk in Critically Ill (NUTRIC) score is effective for screening mechanically ventilated patients. This prospective observational study was conducted to identify nutritional risk in mechanically ventilated patients using a modified NUTRIC (mNUTRIC) score (without using interleukin-6 values).

Methods

All adult patients admitted to the intensive care unit (ICU) for more than 48 hours were included in the study. Data were collected on the variables required to calculate mNUTRIC scores. Patients with mNUTRIC scores ≥5 were considered at high nutritional risk. The assessment data included total ICU length of stay, ventilator-free days, and mortality rates.

Results and conclusion

A total of 75 patients fit the inclusion criteria of the study, including 40 males and 35 females. The mean age was 55.8 years. Forty-five percent of mechanically ventilated patients had mNUTRIC scores ≥5. Mechanically ventilated patients with mNUTRIC scores ≥5 had longer lengths of stay in the ICU (mean ± SD = 11.5±5 days) as compared with 3.5±4 days in patients with mNUTRIC scores ≤4. Moreover, a higher mortality rate (26%) was observed in patients with mNUTRIC scores ≥5. A high mNUTRIC predicted mortality score shows a receiver operating characteristic curve of 0.637 with a confidence interval between 0.399 and 0.875. Forty-five percent of mechanically ventilated patients admitted to the ICU were at nutritional risk, and their mNUTRIC scores were directly related to higher lengths of stay and mortality.

## Introduction

Nutritional support is an essential component of the care of critically ill patients. The prevalence of malnutrition varies between 39% and 50% depending on the screening tool employed and the population studied [1-2]. These nutritional deficiencies are associated with high rates of nosocomial infections, impaired wound healing rates, and high mortality rates [3-4]. The nutritional status of patients admitted to an intensive care unit (ICU) is influenced by both chronic and acute starvation, which can lead to many catabolic processes such as loss of body mass and single and multiple organ failure [5-7].

There is currently little data available on critically ill Pakistani patients. It is important to identify patients who are at risk of malnutrition by assessing their nutritional status within 48 hours of hospital admission. In the hospital setting, various scoring systems, criteria, and tools are used to assess nutritional risk [8-9], including physical examination, dietary intake, severity of illness, functional assessment, and anthropometric data.

However, such assessments are difficult to make in mechanically ventilated and sedated patients. Fluid status and edema can influence changes in weight, as patients may need large-volume resuscitation to maintain their hemodynamics, making it difficult to evaluate muscle and fat wasting. Most of these nutritional assessment tools and criteria do not consider inflammatory processes and hypermetabolic status/muscle wastage in ICU patients [2,10-11].

Based on an assumption that all ICU patients do not have the same nutritional risk, Heyland et al. introduced the Nutrition Risk in Critically Ill (NUTRIC) score. This can be used to identify patients who will benefit from aggressive nutritional support according to their risk of malnutrition [5-6]. In mechanically ventilated patients, nutritional assessment is cumbersome, as their dietary history may be difficult to obtain, and rates of muscle wasting can give a false impression due to edema. Data on nutritional assessment in mechanically ventilated patients using NUTRIC scores are limited [7]. The present study was conducted to identify the prevalence of nutritional risk in mechanically ventilated ICU patients based on modified NUTRIC (mNUTRIC) scores.

## Materials and methods

Patients and methods

A prospective observational study was conducted in the medical intensive care unit (MICU) at Shifa International Hospital, Islamabad, Pakistan, for six months (October 2017–March 2018). Approximately 70 to 80 patients per month are admitted to this MICU. The study was approved by the institutional review board and ethics committee (IRB & EC) of Shifa International Hospital, Islamabad, Pakistan. The approved IRB & EC number of this study was 886-161-2017 and the IRB & EC is in accordance with the International Council for Harmonisation (ICH) and Good Clinical Practice (GCP) guidelines.

Patients aged over 18 years who were admitted to the MICU and remained on a mechanical ventilator for more than 48 hours were included in the study. Patients were excluded if they were: (1) diagnosed as brain dead at admission, (2) were readmitted to the ICU during their same hospital admission, or (3) were transferred to another ICU or hospital. Only patients with a length of stay (LOS) of more than 72 hours were considered for analysis.

mNUTRIC scores (without using interleukin-6 values) were used to identify patients at nutritional risk according to the following five variables: age, number of co-morbidities, days from hospital to ICU admission, and scores on the Acute Physiology and Chronic Health Evaluation II (APACHE II) and Sequential Organ Failure Assessment (SOFA). The scores were based on data obtained during the first 24 hours after MICU admission. Patients with mNUTRIC scores ≥5 were classified as “high,” meaning that they had a higher risk of malnutrition while those with scores ≤4 were considered “low” risk.

The patient data that were collected included data on their demography, the parameters required to calculate mNUTRIC scores, their ICU average length of stay, number of ventilator-free days, and mortality. The data were recorded from patient charts (electronic and/or paper) using a standardized data collection procedure developed for this study (APACHE II and SOFA scores and an mNUTRIC score chart attached to patient records).

The collected data were analyzed with SPSS software (IBM Corp., Statistics for Windows, version 22.0, Armonk, NY, USA). Continuous variables were expressed as means ± standard deviation (SD) and categorical variables were expressed as percentages. Unpaired sample t-tests were used to determine significant differences between bivariate samples in independent groups while Chi-square tests were used to test for significant differences in categorical data. A receiver-operator characteristic curve analysis was used to calculate the sensitivity, specificity, positive predictive value (PPV), and negative predictive value (NPV) of comparisons of outcomes and NUTRIC scores. In all analyses, p = 0.05 was considered statistically significant.

## Results

During the six-month study period, a total of 90 patients were mechanically ventilated for more than 48 hours in the MICU. Fifteen patients were excluded from the study: six were readmitted during the same hospital stay, six were transferred to other ICUs, and three were diagnosed as brain dead at admission. Informed consent was obtained from patient attendants. The data of 75 patients were analyzed. Mean patient age was 55.85 ± 25 years.

**Figure 1:**
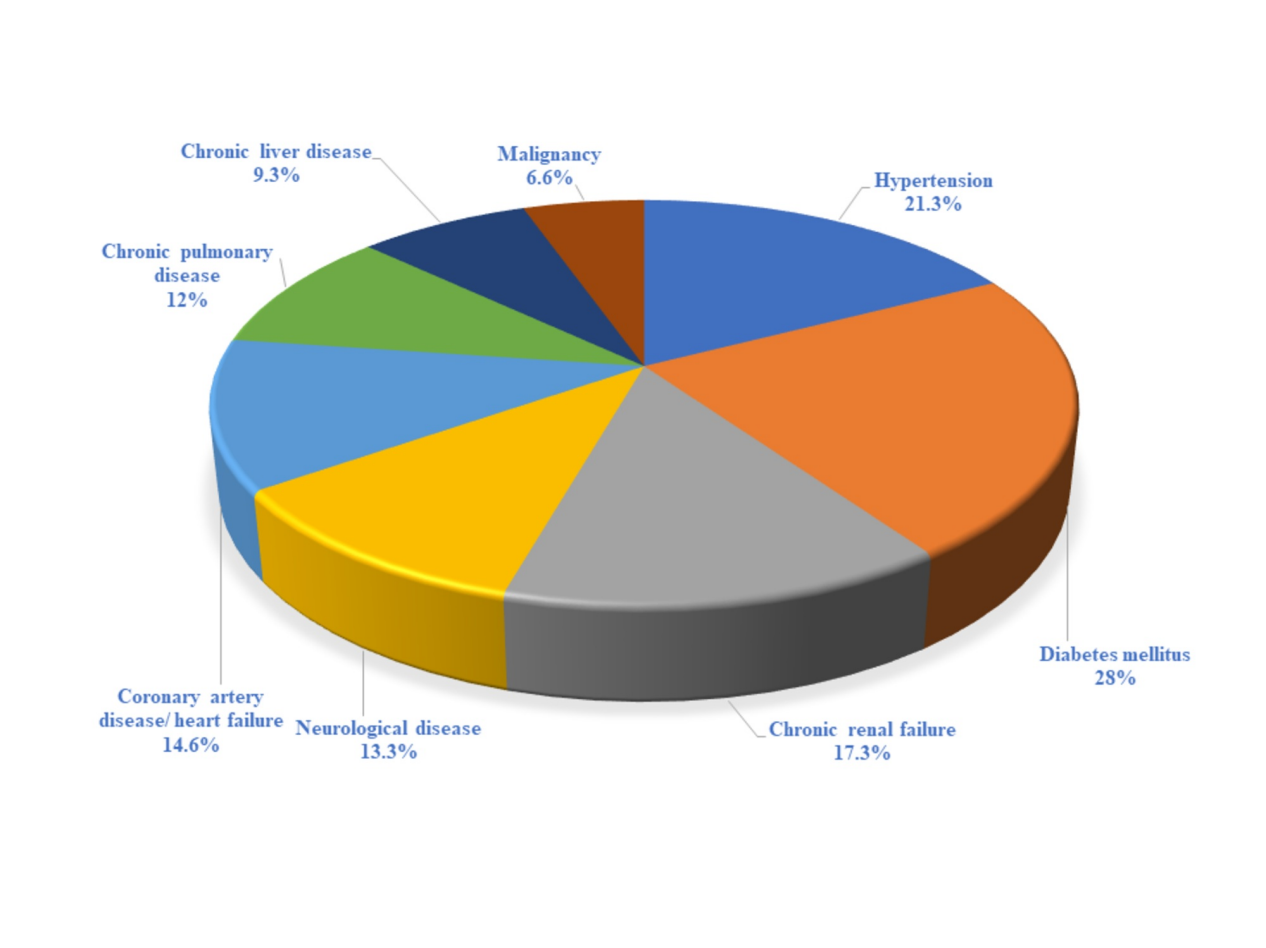
Major comorbidities among the patients studied

Table 1 summarizes the main characteristics of the patients that were included in the study. Of the 75 patients studied, 40 were male and 35 were female. The majority of patients had a medical history including diabetes mellitus (28%), hypertension (21.3%), chronic renal failure (17.3%), and coronary artery disease (14.6%; Figure 1).

**Table 1:** Patient characteristics (n=75) Patient characteristics (n =75)

	n (%)
Male	40 (53.3)
Female	35 (46.6)
Age (years), mean ± SD	55.8 ± 25
Comorbidities	
Hypertension	16 (21.3)
Diabetes mellitus	21 (28)
Chronic renal failure	13 (17.3)
Neurological disease	10 (13.3)
Coronary artery disease/ heart failure	11 (14.6)
Chronic pulmonary disease	09 (12)
Chronic liver disease	07 (9.3)
Malignancy	05 (6.6)

The most common reasons for mechanical ventilation and ICU admission were respiratory failure (30.6%), followed by neurological issues (29.3%), sepsis/shock (26.6%), cardiovascular issues (13.3%), and renal/metabolic issues (12%; Table 2).

**Table 2:** Primary admission diagnosis/ETT indication gastrointestinal (GI); endotracheal tube (ETT)

	n (%)
Respiratory	23 (30.6)
Sepsis/shock	20 (26.6)
Neurological	22 (29.3)
Cardiovascular disease	10 (13.3)
GI/liver disease	03 (4)
Renal/metabolic	09 (12)
Post-operative	03 (4)
Poisoning	02 (2.6)

Table 3 shows a comparative assessment of patients with different nutritional risks based on their mNUTRIC scores. The mean APACHE II and SOFA scores of patients with mNUTRIC scores ≤ 4 were 12.7±4 and 4±6 (p< 0.00), respectively. Patients with mNUTRIC scores ≥ 5 had mean APACHE II and SOFA scores of28.7±6 and 11±7, respectively.

**Table 3:** Comparison of outcomes of patients with different nutrition risk based on mNutric scale. *SOFA = Sequential Organ Failure Assessment, **APACHE-II = Acute Physiology and Chronic Health Evaluation II, ***ICU = Intensive Care Unit modified nutrition risk in critically Ill (NUTRIC); Sequential Organ Failure Assessment (SOFA); Acute Physiology and Chronic Health Evaluation II (APACHE II); intensive care unit (ICU)

	Low nutritional risk (mNUTRIC score ≤ 4)	High nutritional risk (mNUTRIC score ≥ 5)	P-values
mNUTRIC score	30	45	<0.00
Severity of illness			
*SOFA	4±6	11±7	<0.00
**APACHE-II	12.7±4	28.7±6	<0.00
Outcome data			
***ICU length of stay	3.5±4	11.5±5	0.00
Ventilator-free days	1.0±2	5.0±2	0.00
Mortality (%)	3%	26%	-

**Figure 2:**
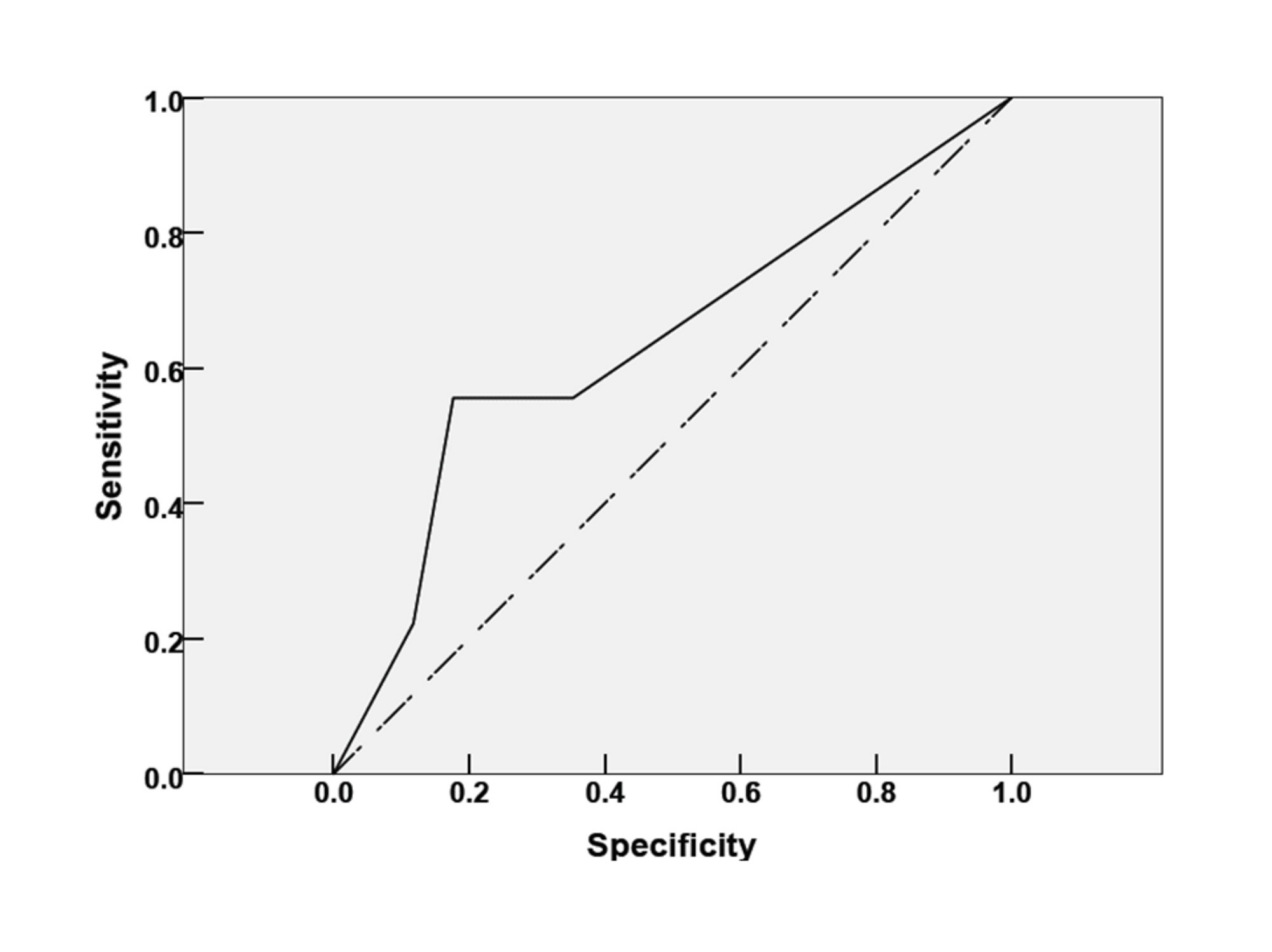
Performance output of the high nutrition risk in critically ill score on a scale of 5–9 to predict ICU mortality in mechanically ventilated patients ICU: intensive care unit

In terms of outcomes for patients with mNUTRIC scores ≤ 4, the mean length of stay and ventilator-free days were 3.5±4 and 1.0±2, respectively, with a 3% mortality rate. However, patients with mNUTRIC scores ≥ 5 had a longer length of stay and a higher mortality rate of 26%. Moreover, mNUTRIC scores ≥ 5 predicted a mortality area of 0.637 under the curve with a confidence interval (CI) of 0.399–0.875 (Figure 2). The calculated PPV and NPV in patients with mNUTRIC scores ≥ 5 were 34.6% and 65.38%, respectively.

## Discussion

The patients admitted to the ICU were critically ill and showed malnutrition criteria on a broader scale. Critical illness with malnutrition in these patients results in an increased occurrence of nosocomial infections, increased hospital stays, difficulty to wean off, and higher morbidity and mortality, with worse functional status at discharge [3,6]. Early nutritional support by an enteral route, if possible, would reduce these complications [12]. Poor nutritional status patients have a poor prognosis, but those with a good nutritional status do not always have better outcomes because of the many other factors associated with their illnesses.

In the present study, 45% of mechanically ventilated patients admitted to the ICU were at high nutritional risk and had mNUTRIC scores ≥ 5. Kalaiselvan et al. [7] reported 42.5% of mechanically ventilated patients to have NUTRIC scores ≥ 5. Similarly, Mendes et al. [13] reported 48.6% of Portuguese ICU patients to be at high nutritional risk. The mortality rate in our study was 26%, which is quite similar to those of other studies such as Kalaiselvan et al. [7], who reported a mortality rate of 31.4% in mechanically ventilated patients. However, a higher mortality rate of > 50% was reported by Moretti et al. [14] in mechanically ventilated patients with similar NUTRIC scores.

There are certain limitations to this study. Firstly, the study was limited to the Pakistani population and had a single-centered design, which might compromise its validity and general applicability. Moreover, the small sample size may limit statistical robustness on a broader scale and cross-comparative studies might not give perfect fittings.

## Conclusions

According to the mNUTRIC scores, 45% of mechanically ventilated patients were at nutritional risk. High mNUTRIC scores were directly proportional to the average length of stay in the ICU and mortality.
